# Biofouling Mitigation by Chloramination during Forward Osmosis Filtration of Wastewater

**DOI:** 10.3390/ijerph15102124

**Published:** 2018-09-27

**Authors:** Takahiro Fujioka, Kha H. Nguyen, Anh Tram Hoang, Tetsuro Ueyama, Hidenari Yasui, Mitsuharu Terashima, Long D. Nghiem

**Affiliations:** 1 Graduate School of Engineering, Nagasaki University, Nagasaki 852-8521, Japan; bb52117705@ms.nagasaki-u.ac.jp (A.T.H.); ueyama@kyowa-kk.co.jp (T.U.); 2 R&D Division, Kyowakiden Industry Co., Ltd., 10-2 Kawaguchi-Machi, Nagasaki 852-8108, Japan; khabruce0202@gmail.com; 3 Faculty of Environmental Engineering, The University of Kitakyushu, 1-1 Hibikino, Wakamatsu, Kitakyushu, Fukuoka 808-0135, Japan; hidenari-yasui@kitakyu-u.ac.jp (H.Y.); m-terashima@kitakyu-u.ac.jp (M.T.); 4 Centre for Technology in Water and Wastewater, University of Technology Sydney, Ultimo NSW 2007, Australia; duclong.nghiem@uts.edu.au

**Keywords:** chloramine, forward osmosis, membrane fouling, pre-concentration, wastewater treatment

## Abstract

Pre-concentration is essential for energy and resource recovery from municipal wastewater. The potential of forward osmosis (FO) membranes to pre-concentrate wastewater for subsequent biogas production has been demonstrated, although biofouling has also emerged as a prominent challenge. This study, using a cellulose triacetate FO membrane, shows that chloramination of wastewater in the feed solution at 3–8 mg/L residual monochloramine significantly reduces membrane biofouling. During a 96-h pre-concentration, flux in the chloraminated FO system decreased by only 6% and this flux decline is mostly attributed to the increase in salinity (or osmotic pressure) of the feed due to pre-concentration. In contrast, flux in the non-chloraminated FO system dropped by 35% under the same experimental conditions. When the feed was chloraminated, the number of bacterial particles deposited on the membrane surface was significantly lower compared to a non-chloraminated wastewater feed. This study demonstrated, for the first time, the potential of chloramination to inhibit bacteria growth and consequently biofouling during pre-concentration of wastewater using a FO membrane.

## 1. Introduction

Anaerobic treatment is a promising platform for simultaneous wastewater treatment and biogas production [1,2,3]. The anaerobic process transforms organic constituents in wastewater to methane gas, which can be used as a renewable fuel to offset the energy footprint of wastewater treatment [4]. Because the typical concentration of organic matter in municipal wastewaters is too low for anaerobic biological treatment, pre-concentration of wastewater (e.g., by 10-fold) is necessary for successful implementation of this process [1]. Studies have demonstrated the potential of forward osmosis (FO) as an effective technology for pre-concentrating wastewater for subsequent anaerobic treatment [5,6,7,8,9]. In the FO process, clean water is drawn from the feed solution (FS), which has a low osmotic pressure, through a semipermeable FO membrane to the draw solution (DS), which has a high osmotic pressure. The FO membrane effectively retains most organic constituents [10,11,12], leading to the pre-concentration of the feed wastewater.

FO is typically operated at a lower flux than the high pressure membrane process (e.g., reverse osmosis); thus, the propensity for membrane fouling is low [11,13,14,15,16]. Due to the complexity of wastewater with high organic matter and microbial contents, membrane fouling remains a major obstacle for sustainable pre-concentration of wastewater using FO. Previous studies have shown that organic matter and microbes in wastewater can readily deposit on the FO membrane surface, which ultimately reduces water flux [17,18,19,20,21,22,23,24,25]. As a result, fouling control is the key to successful pre-concentration of wastewater for FO treatment. Fouling control of a membrane process can be performed by physical and/or chemical membrane cleaning [26]. Physical cleaning includes hydraulic or air scouring with high shear force [27,28,29], osmotic backwashing by a reverse permeate flow [30], and ultrasonication [31,32]. Physical cleaning can be effective during the early stages of biofouling [30], but it is less so after the occurrence of severe fouling [33,34]. Chemical cleaning to remove foulants through the use of chemical reagents is an effective approach to address more permanent or irreversible membrane fouling [35]. Typical reagents for chemical cleaning of cellulose triacetate (CTA)-based FO membranes include ethylenediaminetetraacetic acid (EDTA), sodium hydroxide, and sodium hypochlorite [30,36]. Frequent chemical cleaning can deteriorate performance of any membranes in terms of solute rejection and water permeability and shorten membrane life-time [37]. The pre-concentration of wastewater is likely to undergo severe membrane fouling due to high organics and bacteria contents in the feed, so a fouling mitigation strategy is needed for its effective operation.

A potential approach for mitigating FO membrane fouling is continuous disinfection of the FS (i.e., wastewater) to inhibit microbial activity and alleviate biofouling. Xue et al. [38] reported that continuous chlorination of wastewater effectively slowed the fouling of the FO membrane. Despite the advantage of chlorination as a strong disinfection process, maintaining free chlorine in wastewater requires high doses of chlorine. This is because chlorine is highly reactive and can react with other constituents in wastewater. Thus, free chlorine in wastewater can only be achieved after the chlorine dose has exceeded breakpoint chlorination. In contrast to chlorine, monochloramine is a weaker but more stable disinfectant. The process of disinfection using monochloramine is known as chloramination, which can be readily achieved using a lower chlorine dose (usually in the form of sodium hypochlorite) to react with free ammonia in raw wastewater. Chloramination is commonly used for water recycling applications using a polyamide (PA)-based thin film composite reverse osmosis (RO) membrane [39]. Chloramine is a weak oxidant, and chloramination is an effective biofouling mitigation approach without degrading even PA-based membranes. As a result, chloramination can be applied to both PA and CTA RO membranes. It was hypothesised that chloramination can inactivate bacteria and alleviate membrane biofouling [40,41]. No previous studies have evaluated the effect of chloramination on membrane fouling mitigation in pre-concentrating municipal wastewater using FO membranes.

This study aimed to evaluate the effectiveness of chloramination on fouling mitigation during pre-concentrating municipal wastewater using a CTA FO membrane. Wastewater and artificial seawater were used as the FS and DS, respectively. Chloramination was performed by maintaining monochloramine at 3–8 mg/L in FS. Pre-concentration of wastewater was conducted with or without chloramine, and their fouling levels and membrane morphology were evaluated.

## 2. Materials and Methods

### 2.1. Chemicals

Analytical grade chemicals (NaCl, C_6_H_12_O_6_, KH_2_PO_4_, NH_4_Cl, NaOCl, and NaOH) were purchased from FUJIFILM Wako Pure Chemical Corporation (Osaka, Japan). The draw solution (DS) was prepared at 3.5% (*w*/*w*) NaCl by dissolving NaCl in pure water. Throughout this study, pure water was obtained by purifying tap water using an RO water generation system (RTA-200W, AS ONE, Osaka, Japan). Primary wastewater effluent was collected after the primary sedimentation basin proceeded by grid filtration at a municipal wastewater treatment plant in Nagasaki Prefecture, Japan. The total organic carbon concentration of the primary wastewater effluent was 24 mg/L. Secondary wastewater effluent was collected after activated sludge treatment of the primary wastewater effluent. To accelerate membrane fouling, the wastewater effluent was fortified to simulate the typical organic and nutrient content of high strength wastewater. This was achieved by dissolving C_6_H_12_O_6_, NH_4_Cl, and KH_2_PO_4_ to the primary wastewater effluent at 1000, 305.7, and 175.6 mg/L, respectively, to obtain C:N:P mass ratio of 100:20:10. This primary effluent had a similar ionic composition to that of raw wastewater but did not contain as much suspended solids as raw wastewater. This approach minimised the contribution of the deposition of solid particles on the membrane surface. Thus, observed membrane fouling could be more attributed to the deposition of bacteria and macro-organics on the membrane surface. A monochloramine stock solution was prepared at about 5000 mg/L by mixing a 0.2 M NH_4_Cl solution with a 0.2 M NaOCl solution.

### 2.2. Membrane Treatment System

**Figure 1:**
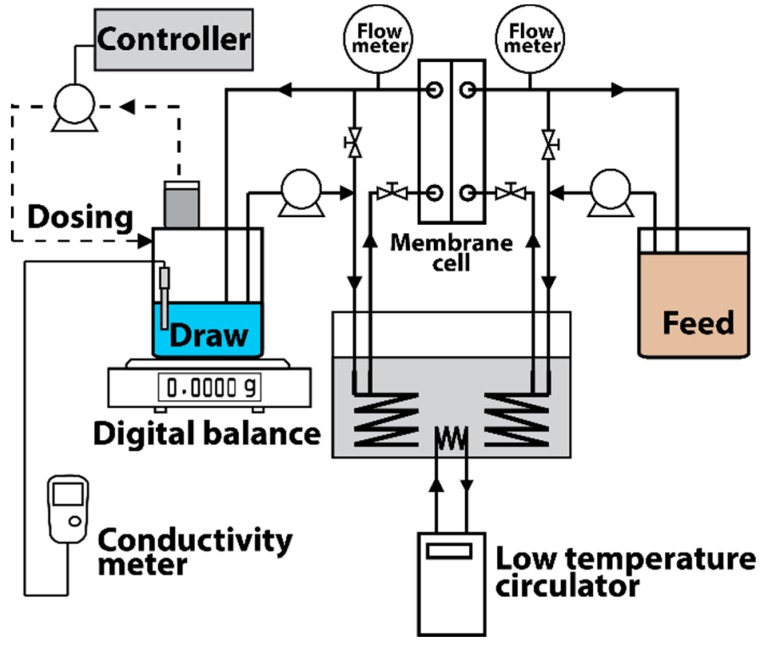
Schematic diagram of the forward osmosis (FO) system.

Flat sheet CTA FO membrane—namely FSB-CTA—was supplied by Fluid Technology Solutions, Inc. (Albany, OR, USA). Unlike polyamide, CTA can be resistant to both chlorine and chloramine. Thus, the CTA FO membrane was selected for extended chloramine exposure and permeance stability for long-term use. For each test, a new CTA FO membrane sample was used. Prior to each experiment, each membrane sample was thoroughly cleared with pure water. The FO membrane set in an acrylic membrane cell (C10-T, Osaka, Nitto Denko, Japan) had an effective membrane area of 60 cm^2^. We used two bench-scale FO systems (Figure 1). The FO system included two diaphragm (membrane) pumps (DCP 8800, Aquatec International, Inc., Irvine, CA, USA) to feed DS and FS solutions, a digital balance (EK-4100i, A&D Company, Tokyo, Japan) to measure the weight of the DS solution, an electromagnetic metering pump (EH-B10VC, IWAKI, Tokyo, Japan) with a control unit for controlling NaCl concentration in the DS, two Styrofoam reservoirs (AS ONE, Osaka, Japan), a low temperature circulator (NCB 2500, EYELA, Tokyo, Japan), and two conductivity meters (AS710, AS ONE, Osaka, Japan).

### 2.3. Experimental Protocol

Each experiment started with pure water flux measurement using pure water in FS and artificial seawater (3.5% NaCl) in DS for 3 h. Thereafter, to confirm stable FO operation, they were operated using the same FS and DS for over 12 h. Prior to the FO treatment test, FS and DS were replaced with 10 L of wastewater and 1 L of new artificial seawater (3.5% NaCl), respectively. During each FO treatment test, the cross flow rate in both FS and DS were maintained at 0.5 L/min, which corresponded to the cross flow velocity of 0.16 and 0.24 m/s in FS and DS, respectively. The temperatures of FS and DS were maintained at 20 °C. Solution pH in the FS was adjusted to approximately 7.5. Conductivity in DS was also maintained at the initial value by automatically dosing the NaCl stock solution using a chemical dose control system. Monochloramine concentration in FS was adjusted every 12 h to maintain a monochloramine concentration of 3–8 mg/L. The tests were conducted for up to 96 h, which was determined to maintain a sufficient volume of feed in the FS reservoir. After 96 h, the pure water flux was measured again using pure water. Surface flushing in FS was applied using pure water at a cross flow rate of 2 L/min for 0.5 h. Pure water flux after the surface flushing was measured using pure water.

### 2.4. Analysis

The concentration of monochloramine in water samples was measured using a portable colorimeter (DR900, Hach, Loveland, CO, USA) with monochlor F reagent pillows. A pH meter (SK-620PH, Sato Keiryoki Mfg. Co., Tokyo, Japan) was used to measure the pH of the FS. Surface images of the FO membrane were attained using a scanning electron microscope (SEM) (Tabletop Microscope TM3030Plus, Hitachi High-Technologies, Tokyo, Japan). Bacterial counts in feed solutions were determined using a fluorescence microscope (Shibasaki, Inc., Chichibu, Japan). Each sample was first diluted 200–20,000 times using microfiltration (MF) membrane-treated pure water. Thereafter, 1 mL of the diluted sample was filtered using a track-etched polycarbonate MF membrane with a 0.2 µm pore size (Meric, Tokyo, Japan). After staining, the bacterial number deposited on 40% of the filter surface area was measured. Total bacterial counts (both viable and nonviable bacteria) were measured by staining with 4′-6-diamidino-2-phenylindole (DAPI) dye (Thermo Fisher Scientific, Waltham, MA, USA). Viable bacterial counts were calculated by deducting counts of dead (inactivated) bacteria determined by staining with 3,6-bis(dimethylamino)acridine hydrochloride solution (Dojindo Laboratories, Kumamoto, Japan) from total bacterial counts.

## 3. Results

### 3.1. Effect of Chloramination

**Figure 2:**
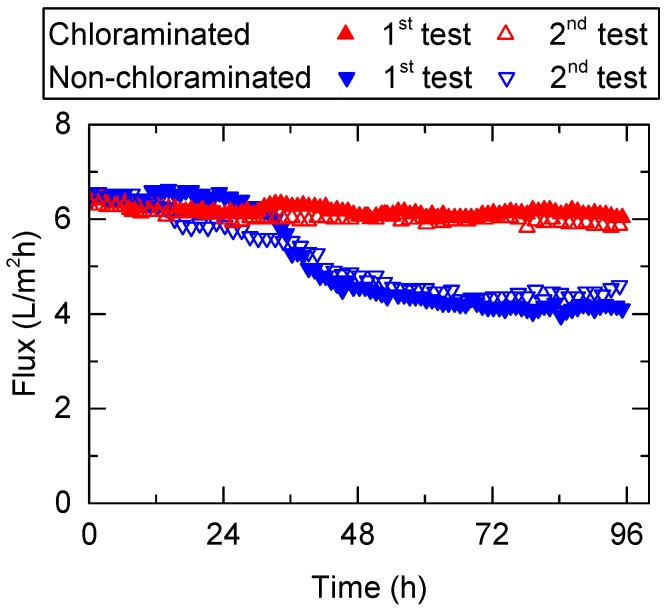
Changes in flux during the pre-concentration of primary wastewater effluent using a FO membrane with and without chloramination (cross flow rate = 0.5 L/min and temperature = 20 °C).

The effect of chloramination in FS on fouling was evaluated by pre-concentrating wastewater via FO with and without chloramination for 96 h. Each test was conducted twice to ensure adequate reproducibility. Overall, a clear difference in the flux profile was observed (Figure 2). The water flux of the non-chloraminated FO system decreased steeply from 6.4 to 4.6 L/m^2^ h from 24 to 48 h during the first test. At the end of the experiment (after 96 h), the flux was 4.1 L/m^2^ h, representing a 35% flux reduction. In contrast, the water flux of the chloraminated FO system only decreased slightly from 6.4 to 6 L/m^2^ h over the same period and the overall flux reduction was 6% during the first test. A similar trend in flux reduction was observed in the replicate experiments (i.e., second test) (Figure 2).

**Figure 3:**
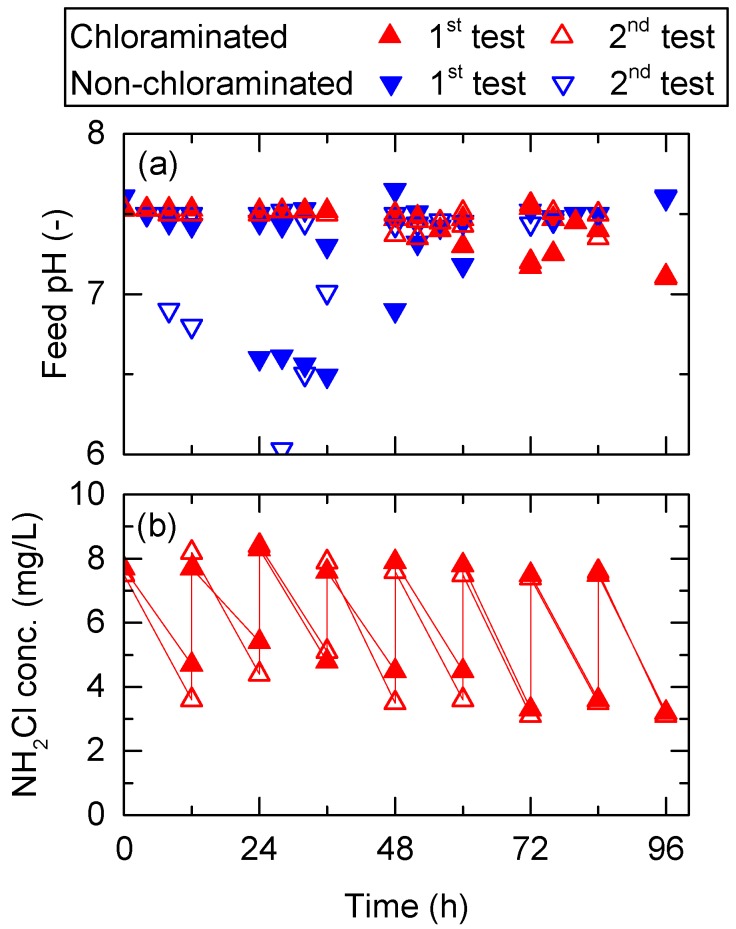
Variation in (**a**) pH and (**b**) monochloramine concentration of the feed solution (FS) during the pre-concentration of primary wastewater effluent using a FO membrane. Feed pH was periodically adjusted to 7.5. NH_2_Cl concentration of FS was adjusted twice per day to achieve 8 mg/L.

**Figure 4:**
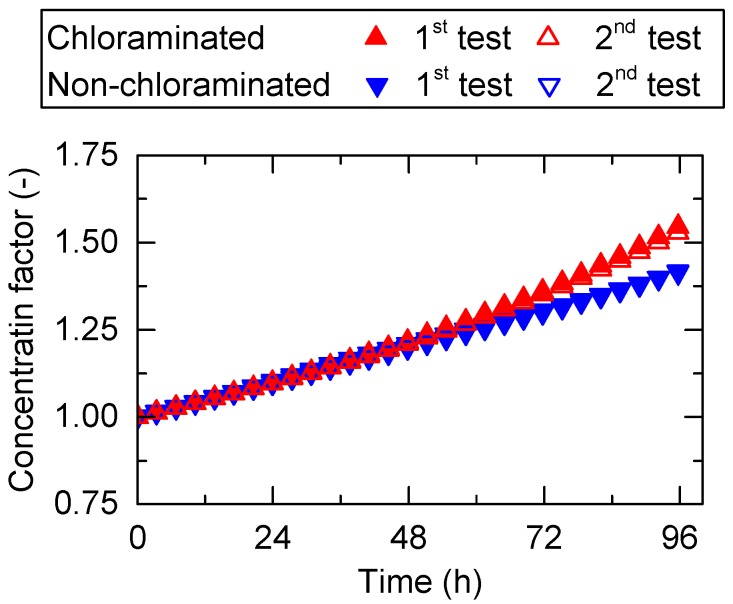
Concentration factor during the pre-concentration of primary wastewater effluent using a FO membrane.

Feed pH in the non-chloraminated FO system decreased considerably over the first 48 h possibly due to the increased carbon dioxide concentrations caused by bacterial growth. Thus, the feed pH in the non-chloraminated FO system was frequently adjusted by dosing with NaOH stock solution. In contrast, the feed pH in the chloraminated FO system remained almost constant at above 7 (Figure 3a) where the dominant species of chloramine was monochloramine. Monochloramine concentration in the chloraminated FO system was maintained between 3 and 8 mg/L during the 96 h experiment (Figure 3b). The concentration of monochloramine in the DS was not detected at the end of FO treatment test in a separate test. Therefore, organics and bacteria in the FS likely consumed most monochloramine through a scavenging process. Notably, chloramine concentration almost halved 12 h after chloramine dosing; thus, the impact of monochloramine in FS on the following process (i.e., anaerobic process) was expected to be low. This will be evaluated on a pilot scale in our future study. Due to the considerable flux reduction in the non-chloraminated FO system, the cumulative volume of water that permeated through the FO membrane was less than in the chloraminated FO system. At the end of the experiment, wastewater with and without chloramination was concentrated by approximately 1.5 and 1.4 times, respectively (Figure 4). The results here indicate that disinfection using monochloramine can effectively mitigate membrane fouling during pre-concentration of wastewater using a FO membrane, achieving a higher concentration in the same period.

### 3.2. Mechanisms of Flux Decline

**Figure 5:**
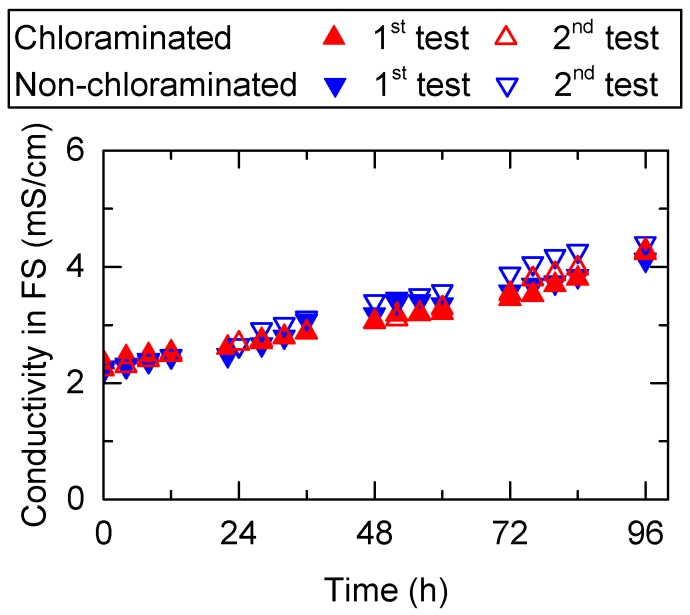
Conductivity in the FS during the pre-concentration of primary wastewater effluent using a FO membrane. Conductivity in the draw solution (DS) was maintained at 47 mS/cm for all tests.

**Figure 6:**
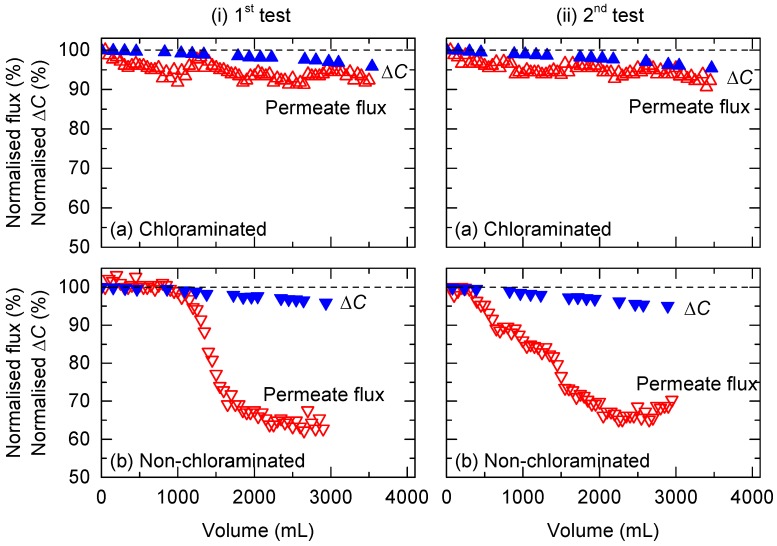
Reduction in normalised flux and conductivity difference between the FS and DS (∆*C*) during the (i) first and (ii) second pre-concentration tests using primary wastewater effluent: (**a**) chloraminated and (**b**) non-chloraminated FO systems.

Flux decline observed during pre-concentration of wastewater was attributed to two factors: (1) reduction in driving force (i.e., osmotic pressure difference between FS and DS) due to the increased salt concentration in FS, and (2) increased hydraulic resistance caused by the deposition of foulants on the membrane surface in the FS. The reduction in the driving force can occur during pre-concentration of wastewater for two reasons: (1) FS is dewatered and concentrated, and (2) back diffusion of salts from DS to FS. For both chloraminated and non-chloraminated FO systems, conductivity in FS increased according to the pre-concentration progress. During the first test, the conductivity of both feed wastewaters increased by approximately 1.8 times from 2.4 to 4.3 mS/cm (Figure 5). The causes of flux decline were further evaluated by tracking changes in conductivity differences between FS and DS, which were considered equivalent to the osmotic pressure difference. Flux in the chloraminated FO system steadily decreased according to the steady decrease in conductivity difference (∆*C*) (Figure 6), suggesting that the reduced osmotic pressure difference was likely the main cause of the reduced flux in the chloraminated FO system. In contrast, the non-chloraminated FO system showed a steep drop in the early stages (Figure 6). The decreased flux at 96 h (30–35%) was far greater than the decreased conductivity difference (∆*C*) (4%). These results suggest that membrane fouling (most likely biofouling) is the dominating cause of flux decline in the non-chloraminated FO system.

**Figure 7:**
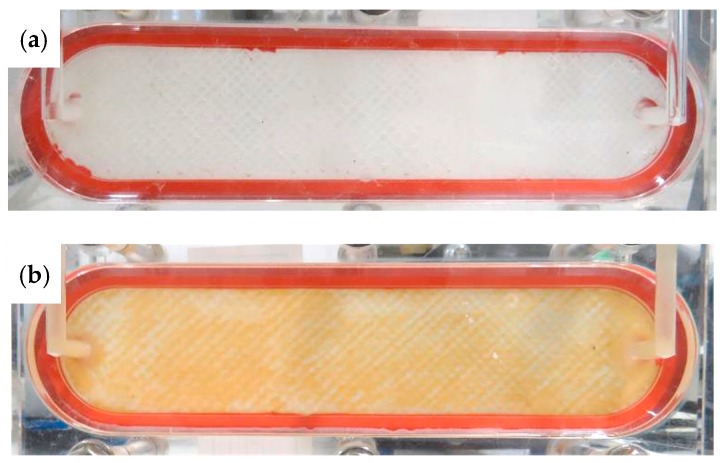
Photos of FO membrane surface (FS side) after 96 h of operation in the first pre-concentration test using primary wastewater effluent: (**a**) chloraminated and (**b**) non-chloraminated.

**Figure 8:**
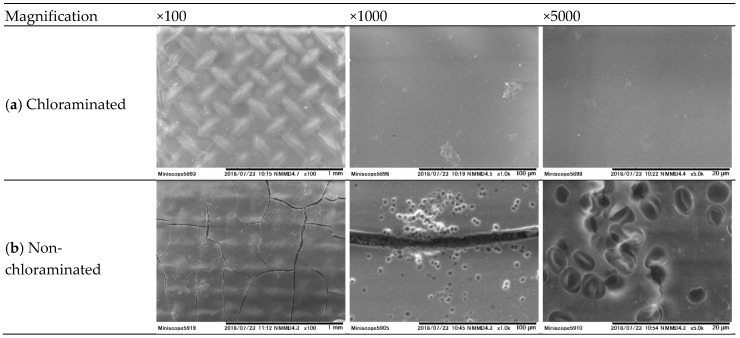
Images of FO membrane surface attained after the first pre-concentration using primary wastewater effluent: (**a**) chloraminated and (**b**) non-chloraminated FO systems.

The deposition of foulants on the FO membrane was evaluated by membrane surface characterisation. The cleanliness of the FO membrane surface in the chloraminated FO system was confirmed through direct visual observation (Figure 7). In contrast, a change in color of the membrane surface in the non-chloraminated FO system was clearly identified. Further analysis of SEM images identified that the FO membrane obtained from the chloraminated FO system held negligible amounts of substances deposited on the membrane surface at any magnification (Figure 8a), which were almost identical to a virgin FO membrane (image not shown). In contrast, the SEM image of the FO membrane attained from the non-chloraminated FO system revealed the formation of a cake layer with some cracks at a magnification of 100×. Other SEM images at 1000 and 5000× magnification also showed a deposition of many spherical particles of approximately 4 µm in diameter, which were likely spherical bacteria (e.g., coccus) (Figure 8b).

**Figure 9:**
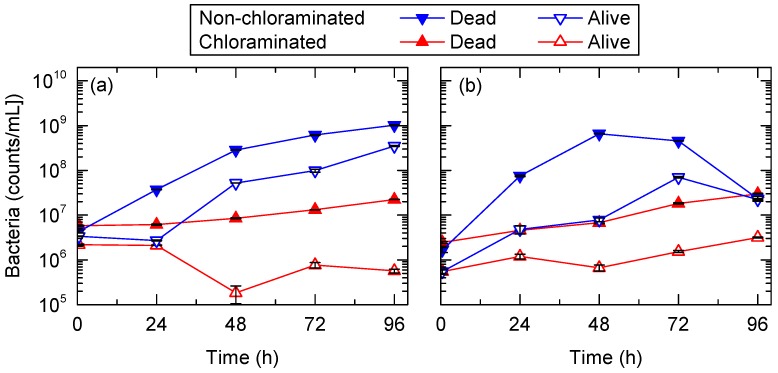
Dead and alive bacterial counts in the chloraminated and non-chloraminated FO systems during the (**a**) first and (**b**) second pre-concentration tests using primary wastewater effluent. Error bars show the standard deviation of two replicate experiments.

To evaluate the impact of bacteria in the FS on membrane fouling, counts of dead and alive bacteria were measured every 24 h. Overall, bacterial counts in the non-chloraminated FO system were higher than in the chlorinated FO system in all experiments. For example, dead bacterial counts in the non-chloraminated FO system increased by almost 240 times, from 4 × 10^6^ to 1 × 10^9^ counts/mL, at the end of the first test (Figure 9). Similarly, alive bacterial counts in the non-chloraminated FO system increased according to the progress of the FO treatment. The increase in bacterial counts from 24 to 48 h was considerable, which was in line with the steep decrease in flux, as observed in Figure 2. Bacterial counts at the later stage of the second pre-concentration test considerably decreased after 72 h (Figure 9b). This may be attributed to the depletion of nutrients in the FS of the non-chloraminated FO system and bacteria inactivation. In fact, the flux did not recover after 72 h (Figure 2). In contrast, over the same test period, dead and alive bacterial counts in the chloraminated FO system showed a minor change from 6 × 10^6^ to 2 × 10^7^ counts/mL and 2 × 10^6^ to 6 × 10^5^ counts/mL, respectively, during the first test. Bacteria produce extracellular polymeric substances composed of polysaccharides and proteins, and they have been frequently reported to cause major membrane biofouling in wastewater treatment [42]. Although there is no direct evidence that these dead bacteria contributed to membrane fouling, the large difference in bacterial counts in the FS (Figure 9) and on the membrane surface (Figure 8) between chloraminated and non-chloraminated FO indicate that bacterial growth could be the major cause of membrane fouling in the pre-concentration of wastewater. In addition, the results indicated that membrane biofouling during wastewater pre-concentration could be minimised by applying chlorination to the feed solution. Further research is recommended to quantify the contribution of bacterial deposition to membrane fouling.

### 3.3. Fouling Reversibility

**Figure 10:**
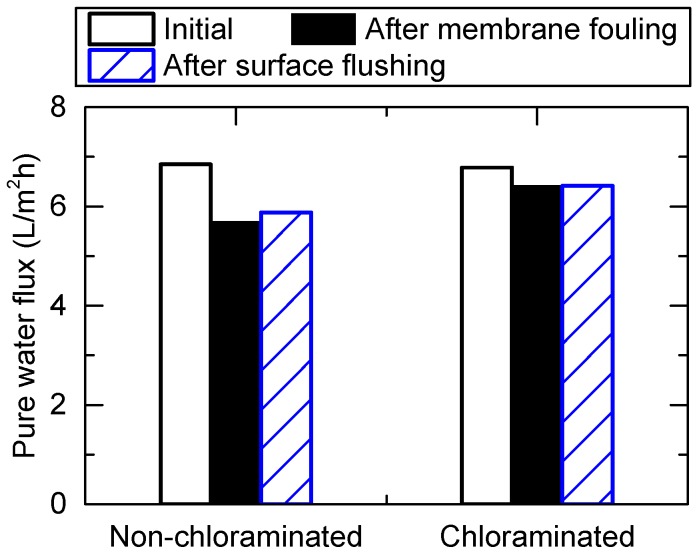
Pure water flux before and after membrane fouling, and surface flushing at the second test using primary wastewater effluent.

Fouling reversibility was evaluated by measuring the pure water flux before and after the FO treatment of wastewater, and surface flushing during the second test of this study. In the non-chloraminated FO system, the FO treatment of the wastewater caused a flux reduction from 6.9 to 5.7 L/m^2^ h (Figure 10). The following surface flushing at a cross-flow rate of 2 L/min applied for 0.5 h only resulted in a slight flux recovery to 5.9 L/m^2^ h from 5.7 L/m^2^ h. These results suggest the presence of a biofilm layer on the membrane surface. Unlike organic and colloidal fouling, this form of fouling is not readily reversible by physical surface flushing. With chloramination, the flux only slightly reduced from 6.9 to 6.4 L/m^2^ h, representing a 6% flux decrease (Figure 10). A similar observation can be made for the chloraminated FO system, as surface flushing at a cross-flow rate of 2 L/min did not result in any discernible flux recovery. Similar reversibility was also confirmed with a separate test using the secondary wastewater effluent (Figure S1). These results highlight the importance of fouling prevention by chloramination as biofouling cannot be controlled by hydraulic flushing.

## 4. Conclusions

The efficacy of chloramination on biofouling mitigation of a cellulose triacetate FO membrane during pre-concentration of wastewater was demonstrated. Without chloramination, pre-concentration of wastewater by FO for 96 h resulted in a 35% flux reduction. In contrast, chloramination with a residual monochloramine of 3–8 mg/L in the FS led to a flux reduction of only 6% and this flux reduction was attributed to the increase in feed solution salinity. SEM analysis showed that the FO membrane surface in the chloraminated FO system had less deposition of bacteria-like particles. Bacterial counts also confirmed that the FS in the chloraminated FO system resulted in less bacterial deposition on the membrane surface. Overall, this study demonstrated that chloramination can inhibit bacterial growth during pre-concentration of wastewater using a FO membrane, effectively mitigate biofouling. Because the continuous addition of chloramine to the FS causes an increase in capital and operating costs, economic evaluation at the pilot-scale is required to scale up to a full-scale system.

## Supplementary Materials

The following are available online at http://www.mdpi.com/1660-4601/15/10/2124/s1, Figure S1: (a) Changes in flux during the pre-concentration of secondary wastewater effluent by FO membrane with and without chloramination, and (b) pure water flux before and after membrane fouling, and surface flushing.
