# Supplementary material for: Biofouling Mitigation by Chloramination during Forward Osmosis Filtration of Wastewater
Source: Int J Environ Res Public Health. 2018 Sep 27;15(10):2124. doi: 10.3390/ijerph15102124 (PMC6210331; doi:10.3390/ijerph15102124)
Supplement: Supplementary file 1 [file ijerph-15-02124-s001.pdf]

International Journal of
Environmental Research and Public Health

Supplementary Material

Biofouling Mitigation by Chloramination during Forward Osmosis Filtration of Wastewater

Takahiro Fujioka, Kha H. Nguyen, Anh Tram Hoang, Tetsuro Ueyama, Hidenari Yasui, Mitsuharu Terashima, and Long D. Nghiem

(a)

(b)

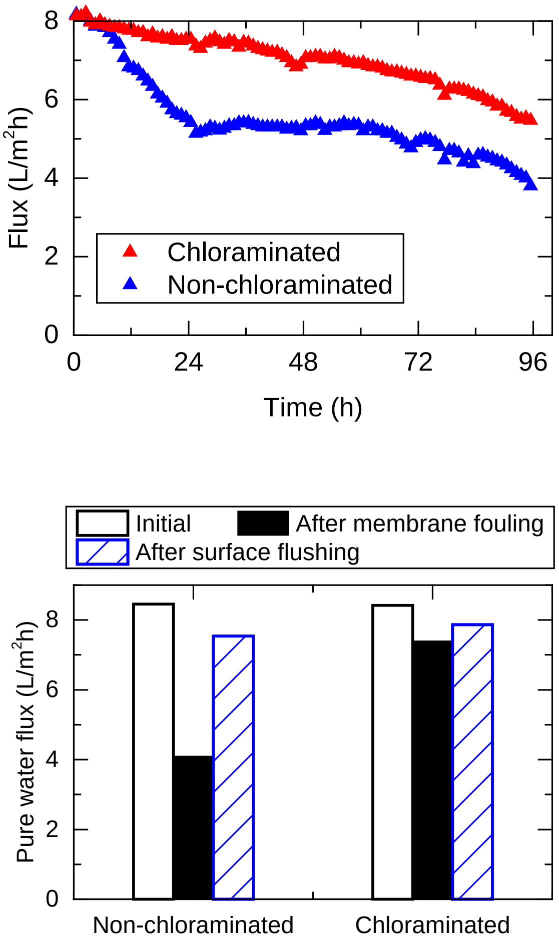

Figure S1. (**a**) Changes in flux during the pre-concentration of secondary wastewater effluent by FO membrane with and without chloramination, and (**b**) pure water flux before and after membrane fouling, and surface flushing.
